# Mechanistic insights into the anti-depressant effect of emodin: an integrated systems pharmacology study and experimental validation

**DOI:** 10.18632/aging.203072

**Published:** 2021-05-29

**Authors:** Peng Zeng, Xiao-Ming Wang, Chao-Yuan Ye, Hong-Fei Su, Ying-Yan Fang, Teng Zhang, Qing Tian

**Affiliations:** 1 Department of Pathology and Pathophysiology, School of Basic Medicine, Tongji Medical College, Key Laboratory of Neurological Disease of National Education Ministry, Huazhong University of Science and Technology, Wuhan 430030, China; 2 Hubei Key Laboratory for Kidney Disease Pathogenesis and Intervention, Hubei Polytechnic University School of Medicine, Huangshi 435000, China; 3 Department of Neurology, Shanxian Central Hospital, The Affiliated Huxi Hospital of Jining Medical College, Heze 274300, China

**Keywords:** emodin, depression, systems pharmacology, neuroinflammation, PI3K-Akt signaling pathway

## Abstract

Depression is a complex neuropsychiatric disease involved multiple targets and signaling pathways. Systems pharmacology studies could potentially present a comprehensive molecular mechanism to delineate the anti-depressant effect of emodin (EMO). In this study, we investigated the anti-depressant effects of EMO in the chronic unpredictable mild stress (CUMS) rat model of depression and gained insights into the underlying mechanisms using systems pharmacology and molecular simulation analysis. Forty-three potential targets of EMO for treatment of depression were obtained. GO biological process analysis suggested that the biological functions of these targets mainly involve the regulation of reactive oxygen species metabolic process, response to lipopolysaccharide, regulation of inflammatory response, etc. KEGG pathway enrichment analysis showed that the PI3K-Akt signaling pathway, insulin resistance, IL-17 signaling pathway were the most significantly enriched signaling pathways. The molecular docking analysis revealed that EMO might have a strong combination with ESR1, AKT1 and GSK3B. Immunohistochemical staining and Western blotting showed that 2 weeks’ EMO treatment (80 mg/kg/day) reduced depression related microglial activation, neuroinflammation and altered PI3K-Akt signaling pathway. Our findings provide a systemic pharmacology basis for the anti-depressant effects of EMO.

## INTRODUCTION

Depression is a global health concern, affecting approximately 6% of the world population each year [1]. As estimated by the World Health Organization, depression was the second most prevalent disease through 2020 [2]. Depression not only decreases the quality of life in depression patients but also brings serious economic burden on society [3]. Several theories have been proposed to account for depression, including the monoamine hypothesis [4], the hypothalamic-pituitary-adrenal (HPA) axis hyperactivity hypothesis [5], the neural plasticity hypothesis [6] and the neurodegeneration hypothesis [7]. Although many possible theories have been proposed, the treatment of depression is still not optimistic [8, 9]. It takes several weeks for anti-depressants to demonstrate full effectiveness and often occurring adverse effects, leading to poor compliance [10]. Fluoxetine (Prozac), for instance, a selective serotonin reuptake inhibitor, has obvious side effects result in acute nausea, headaches, weight gain and sexual dysfunction [8]. Therefore, search for more effective and reliable anti-depressants is essential.

Emodin (EMO) is an anthraquinone isolated from *Rheum officinale*, known for its pleiotropic pharmacological actions, including anti-inflammation, anti-oxidant, anti-cancer and anti-virus effects [11–14]. Mounting evidence suggests that EMO plays a protective role in brain diseases, such as ischemic stroke, hemorrhagic stroke, traumatic brain injury, tumors, Alzheimer’s disease, depression and others [13, 15–17]. EMO ameliorates chronic unpredictable mild stress (CUMS) induced depression-related behaviors by altering the glucocorticoid receptor (GR) and brain-derived neurotrophic factor (BDNF) levels in the hippocampus [16]. Although the anti-depressant effect of EMO was reported in a few articles, the underlying molecular mechanism remains largely unclear.

As a powerful tool for drug discovery and development, systems pharmacology approach combines network biology and multipharmacology [18]. To investigate the precise pharmacological mechanism of EMO against depression, an integrated systems pharmacology approach and molecular docking were employed. In this study, we first investigated the anti-depressant effects of EMO using the well-validated and widely used CUMS model of depression [19, 20]. The sucrose preference test (SPT), open field test (OFT) and forced swimming test (FST) were performed to evaluate depressive-like behaviors in rats. Then, a systems pharmacology approach was used to uncover the molecular mechanism of EMO against depression, to identify the main anti-depressant signaling pathways and to provide a valuable theoretical basis for clinical application.

## RESULTS

### EMO improves CUMS induced depression-related behaviors

**Figure 1:**
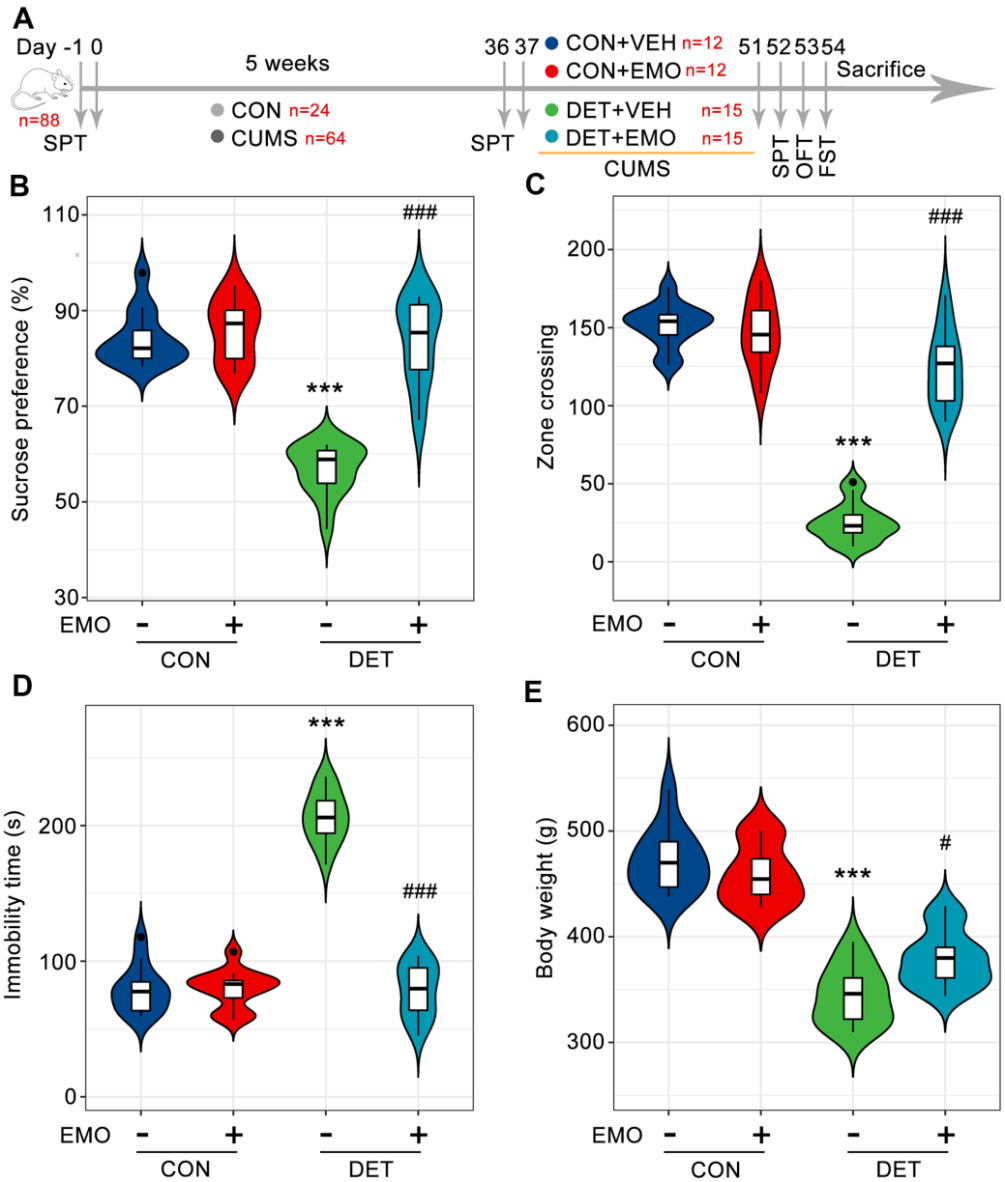
**EMO improves CUMS induced depression-related behaviors.** (**A**) Diagram of the experimental workflow. Twenty-four rats were randomly chosen as the control (CON) group, and 64 rats were exposed to 5 weeks of CUMS. On day 36, all rats underwent SPT to evaluate their status. Depressive-tendency rats (DET, n = 30) were defined as those with a more than a 20% decrease in sucrose water intake, and then divided into two groups: the DET+vehicle (VEH) group and DET+EMO (80 mg/kg/day) group (n = 15/group). After two weeks of EMO treatment, the animals completed the SPT, OFT and FST. (**B**) Percentage of sucrose water consumed in the SPT at day 52. The numbers of zone crossings in the OFT (**C**) and the immobility times in the FST (**D**) were recorded. (**E**) The body weight was measured at day 51. Data were expressed as the means ± SEM. *** *p* < 0.001 DET+VEH vs CON+VEH. # *p* < 0.05, ### *p* < 0.001 DET+EMO vs DET+VEH.

To assess the anti-depressant effect of EMO, a CUMS induced depression model was employed [19–21]. As shown in Figure 1A, after exposure to CUMS for 5 weeks, 30 out of 64 rats were defined as depressive tendency rats (DET rats, reduced the sucrose water intake by more than 20%). After 2 weeks of EMO or vehicle treatment, depressive-like symptoms were evaluated by the SPT, FST, OFT and body weight. The sucrose preference percentage of DET+VEH rats (84.2 ± 1.7) was significantly reduced compared with that of CON+VEH rats (56.8 ± 1.4). However, the sucrose preference percentage of DET+EMO rats (83.6 ± 2.3) treated for 2 weeks with EMO significantly increased compared with DET+VEH rats (Figure 1B). In the OFT, the number of zone crossings in DET+VEH rats (25 ± 3.1) was decreased compared with that in CON+VEH rats (151.5 ± 4.0), whereas treatment with EMO led to a significant increase (124.1 ± 6.2) (Figure 1C). The results of FST revealed that CUMS exposure (206.3 ± 4.9 s) significantly increased immobility times compared with CON+VEH (78.9 ± 5.0 s), while EMO at dose of 80 mg/kg/day treatments (78.5 ± 5.0 s) significantly reduced immobility times versus DET+VEH (Figure 1D). CUMS exposure caused a significant reduction in body weight in DET+VEH rats (344.1 ± 7.0 g) relative to CON+VEH rats (472 ± 8.6 g), and the abnormal decreases in body weight were normalized by EMO treatment (Figure 1E). These results indicate that 2 weeks’ EMO treatment could ameliorate the depressive-like behaviors induced by CUMS.

### Gene ontology (GO) biological process and the kyoto encyclopedia of genes and genomes (KEGG) pathway enrichment analyses of depression-related targets

**Figure 2:**
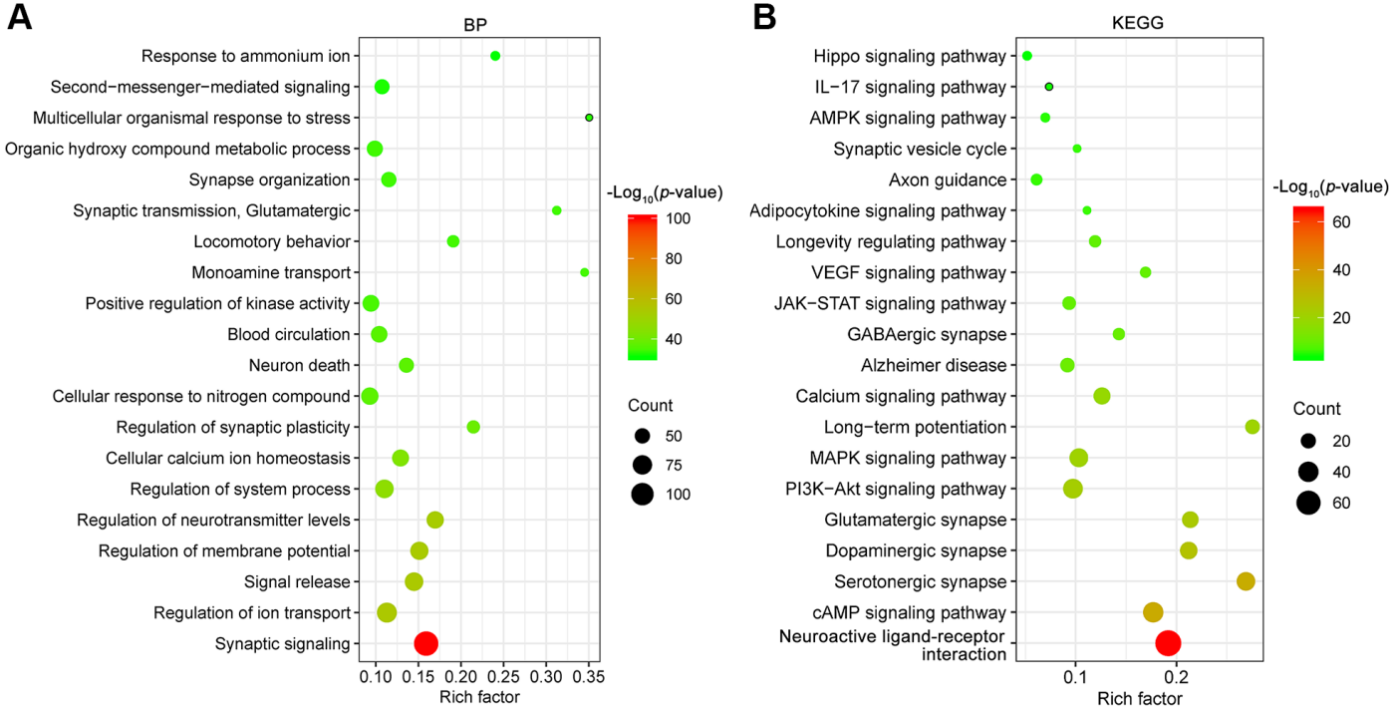
GO biological process (BP, **A**) and KEGG (**B**) pathway enrichment analysis of depression targets. The X-axis represents the rich factor, bubble size represents the count of targets enriched in terms and the color represents the *p* value.

To better understand the underlying mechanisms of depression, a total of 340 depression candidate targets were obtained from the Therapeutic Target Database (TTD) [22], GeneCards and Rat Genome Database. Next, GO biological process and KEGG pathway enrichment analyses were carried out using Metascape [23]. The primary enriched GO biological process terms were synaptic signaling (GO:0099536), regulation of ion transport (GO:0043269), signal release (GO:0023061), regulation of membrane potential (GO:0042391), regulation of neurotransmitter levels (GO:0001505), regulation of system process (GO:0044057) and so on (Figure 2A). Among these terms, synaptic signaling (GO:0099536) exhibited the highest number of target connections (degree = 118), followed by regulation of ion transport (GO:0043269, degree = 80).

Furthermore, the enrichment analysis of KEGG pathways included 326 pathways (*p* < 0.01). The top 20 enriched KEGG pathways are presented in Figure 2B. The KEGG pathways involved were mainly the neuroactive ligand-receptor interaction (hsa04080), cAMP signaling pathway (hsa04024), serotonergic synapse (hsa04726), dopaminergic synapse (hsa04728), glutamatergic synapse (hsa04724), PI3K-Akt signaling pathway (hsa04151), MAPK signaling pathway (hsa04010), long-term potentiation (hsa04720), calcium signaling pathway (hsa04020), Alzheimer disease (hsa05010) and soon. In particular, there were 37 targets involved in the PI3K-Akt signaling pathway (hsa04151), and the associated targets included AKT1, BDNF, GDNF, GSK3B, PTEN, TP53, VEGFA, etc. The above results pointed out that multiple mechanisms were involved in the pathogenesis of depression. Therefore, drugs with multiple anti-depressant targets may be attractive antidepressants.

### Pharmacological and molecular properties of EMO

Compounds that complied with the requirements of Lipinski’s rule of five seem to be more likely to become drugs. SwissADME prediction [24, 25] showed that the EMO satisfied Lipinski’s rule of five (molecule weight (MW): 270.24 g/mol, lipid-water partition coefficient (log P): 2.72, hydrogen bond donors (Hdon): 3, hydrogen bond acceptors (Hacc): 5, rotatable bonds (Rbon): 0. Other chemical and pharmacological properties of this EMO were also evaluated, including topological polar surface area (TPSA) = 94.83 Å and solubility (Log S) = -3.67. Furthermore, the drug-likeness weight of EMO is 0.683, which is obtained by searching the Encyclopedia of Traditional Chinese Medicine (ETCM, http://www.tcmip.cn/ETCM/) [26]. In general, molecules with drug-likeness weight ≥ 0.18 have good drug likeness. Further search of admetSAR database (http://lmmd.ecust.edu.cn/admetsar2/) [27] showed that the human oral bioavailability probability of EMO was 0.6. These results indicate that EMO has acceptable pharmacokinetic properties.

### Shared targets between EMO-related targets and depression-related targets

**Figure 3:**
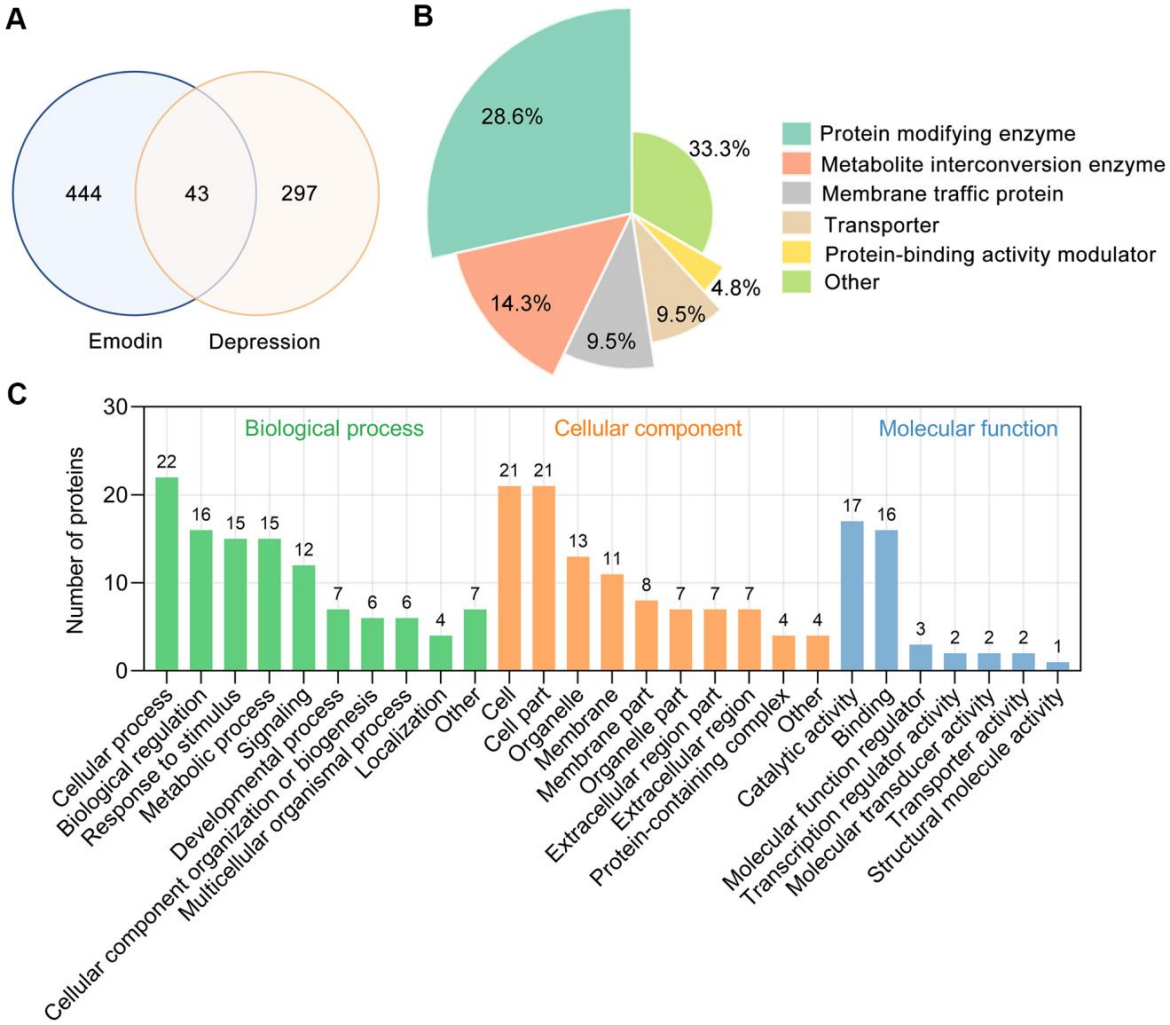
**Bioinformatics analysis of target proteins of EMO against depression.** (**A**) Venn diagram was used to determine the intersection between the EMO and depression targets. (**B**) Panther classification categorized target proteins of EMO against depression. The figures next to the pie chart indicate the percentage of the protein in the given functional class. (**C**) GO classification of targets of EMO against depression at GO level 2 into 3 categories: biological process, molecular function and cellular component.

By retrieving the Pathway Assembly from Literature Mining-an Information Search Tool (PALM-IST) database [28] and validated in the PubMed database, a total of 487 potential targets of EMO were obtained. From the TTD, GeneCards and Rat Genome Database, 340 depression-related targets were identified in total. Taking the intersection of the potential targets of EMO and depression, 43 potential targets were screened out (Figure 3A). Detailed information about common targets is provided in Table 1.

**Table 1:** The target information of EMO against depression.

**Number**	**Gene ID**	**Gene symbol**	**Description**
1	5243	ABCB1	ATP binding cassette subfamily B member 1
2	1636	ACE	angiotensin I converting enzyme
3	207	AKT1	AKT serine/threonine kinase 1
4	213	ALB	albumin
5	1302	COL11A2	collagen type XI alpha 2 chain
6	1385	CREB1	cAMP responsive element binding protein 1
7	1401	CRP	C-reactive protein
8	1493	CTLA4	cytotoxic T-lymphocyte associated protein 4
9	1557	CYP2C19	cytochrome P450 family 2 subfamily C member 19
10	1559	CYP2C9	cytochrome P450 family 2 subfamily C member 9
11	1621	DBH	dopamine beta-hydroxylase
12	2099	ESR1	estrogen receptor 1
13	2260	FGFR1	fibroblast growth factor receptor 1
14	2263	FGFR2	fibroblast growth factor receptor 2
15	2736	GLI2	GLI family zinc finger 2
16	2796	GNRH1	gonadotropin releasing hormone 1
17	2932	GSK3B	glycogen synthase kinase 3 beta
18	3479	IGF1	insulin like growth factor 1
19	3586	IL10	interleukin 10
20	3553	IL1B	interleukin 1 beta
21	3558	IL2	interleukin 2
22	3569	IL6	interleukin 6
23	3630	INS	insulin
24	3845	KRAS	KRAS proto-oncogene, GTPase
25	3930	LBR	lamin B receptor
26	5604	MAP2K1	mitogen-activated protein kinase kinase 1
27	5605	MAP2K2	mitogen-activated protein kinase kinase 2
28	5594	MAPK1	mitogen-activated protein kinase 1
29	4137	MAPT	microtubule associated protein tau
30	5027	P2RX7	purinergic receptor P2X 7
31	11315	PARK7	Parkinsonism associated deglycase
32	5144	PDE4D	phosphodiesterase 4D
33	5728	PTEN	phosphatase and tensin homolog
34	6622	SNCA	synuclein alpha
35	6647	SOD1	superoxide dismutase 1
36	282974	STK32C	serine/threonine kinase 32C
37	6855	SYP	synaptophysin
38	7020	TFAP2A	transcription factor AP-2 alpha
39	7124	TNF	tumor necrosis factor
40	7157	TP53	tumor protein p53
41	1890	TYMP	thymidine phosphorylase
42	7422	VEGFA	vascular endothelial growth factor A
43	7494	XBP1	X-box binding protein 1

Forty-three potential targets of EMO against depression were categorized into 6 different classes based on their cellular function, of which protein modifying enzyme (PC00260, 28.6%) was the most enriched class (Figure 3B). Among these protein modifying enzymes, AKT1, GSK3B, MAPK1 and STK32C belong to non-receptor serine/threonine protein kinases, PTEN belongs to protein phosphatases and ACE belong to metalloprotease. In addition, 14.3% of the common targets are involved in metabolite interconversion enzyme (PC00262, Figure 3B). The above results suggested that EMO can exert an anti-depressant role through multiple targets and biological functions.

To obtain an overview of the 43 potential targets of EMO against depression, GO functional classification was investigated. Most of the potential targets existed on the cell and cell part with catalytic activity. Within the biological processes, the majority of potential targets were enriched in cellular process (GO:0009987), biological regulation (GO:0065007), response to stimulus (GO:0050896) and metabolic process (GO:0008152) (Figure 3C). These results indicate that EMO has multiple synergistic effects in biological processes.

### Protein–protein interaction (PPI) analysis of targets of EMO against depression

**Figure 4:**
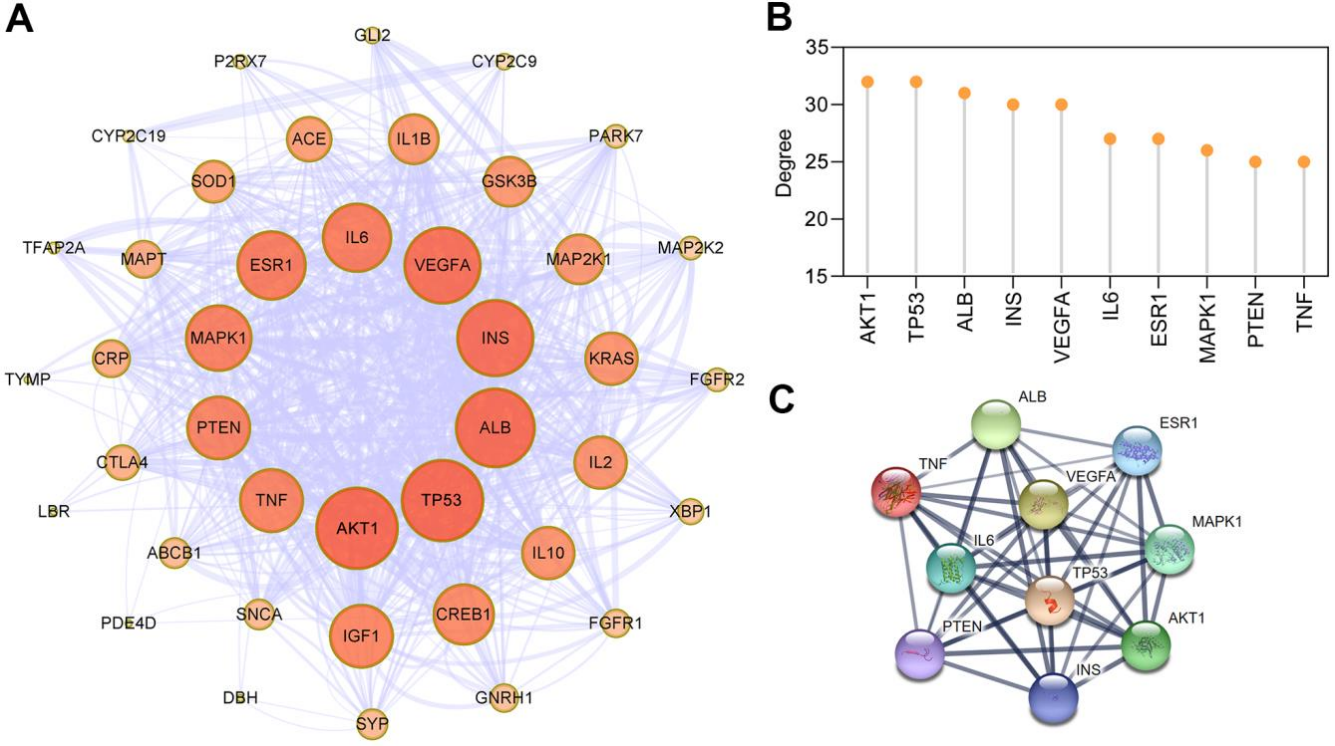
**PPI network construction for targets of EMO against depression.** (**A**) PPI network of EMO against depression. Nodes represent target proteins and edges represent interactions among targets. The darker the color and the larger the node, the higher the degree. The thickness of the edges represents the combined score. (**B**) The top 10 core targets were ranked by degree. (**C**) PPI network of core targets extracted from (**A**).

To explore the interaction effect between 43 potential targets of EMO against depression, PPI analysis was performed using the STRING 11.0 database [29]. In the PPI network, a total of 43 nodes and 332 edges were acquired, and the average node degree was 15.4 (Figure 4A). The larger the node and the darker the color, the greater the degree value was. AKT1, TP53, ALB, INS, VEGFA, IL6, ESR1, MAPK1, PTEN and TNF, which are ranked by degree, were identified as core targets (Figure 4B). Among these, AKT1 showed the highest degree (32). These core targets formed a complex PPI network, included 10 nodes and 45 edges, and the average node degree was 9 (Figure 4C). This demonstrates that these core targets are closely related to other targets in the PPI network, suggesting that these targets may play a key role in depression treatment.

### Potential synergistic mechanisms of EMO against depression

### GO biological process enrichment analysis

**Figure 5:**
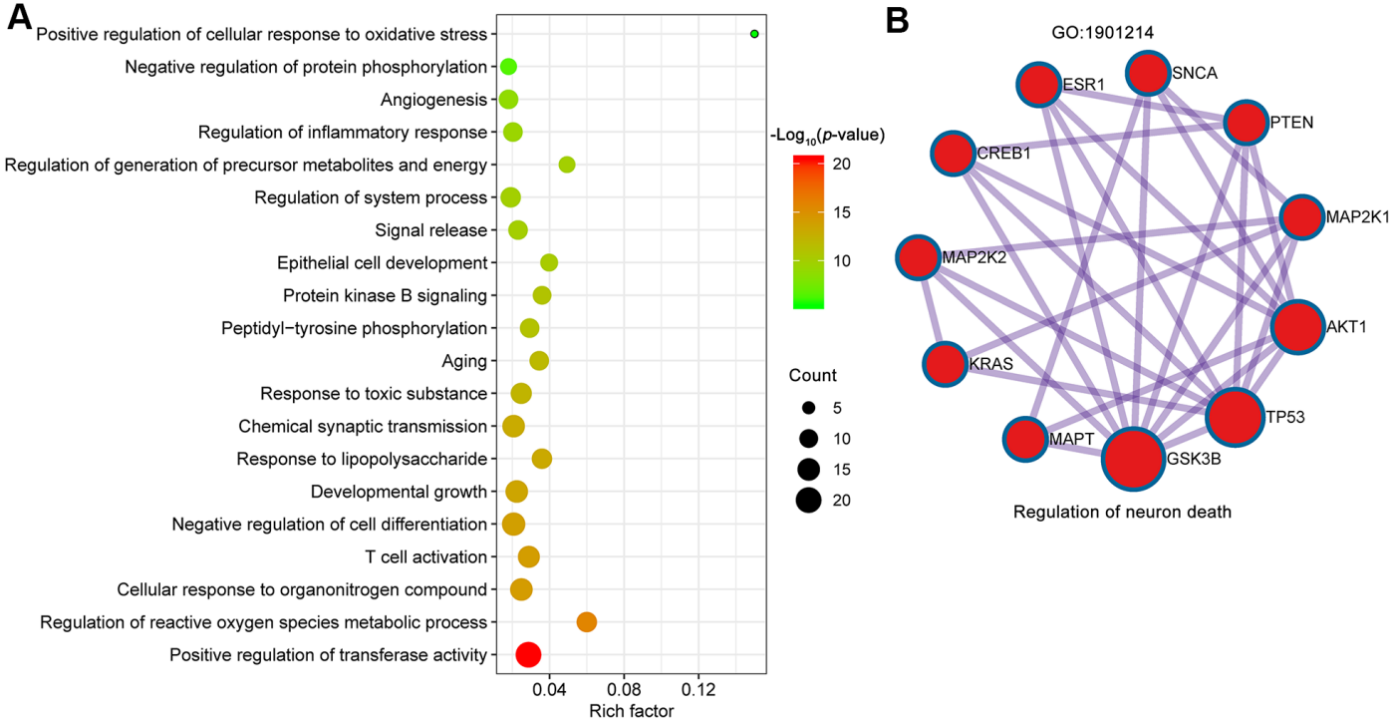
**Biological processes of GO enrichment analysis.** (**A**) Top 20 bubble chart of biological process of GO enrichment analysis. The X-axis represents the rich factor, bubble size represents the count of targets enriched in terms and the color represents the *p* value. (**B**) The regulation of neuron death (GO:1901214) was identified by MCODE algorithm.

The common targets were further analyzed for functional prediction by Metascape. The primary enriched GO biological process was positive regulation of transferase activity (GO:0051347), regulation of reactive oxygen species metabolic process (GO:2000377), cellular response to organonitrogen compound (GO:0071417), T cell activation (GO:0042110), negative regulation of cell differentiation (GO:0045596) and so on (Figure 5A). The Molecular Complex Detection (MCODE) algorithm (k-core = 2) was further used to identify highly interconnected clusters, and regulation of neuron death (GO: 1901214) was identified (score = 5.6) (Figure 5B). In particular, 7 out of 10 proteins involved in regulation of neuron death were core targets (AKT1, TP53, INS, VEGFA, IL6, MAPK1, PTEN and TNF).

### KEGG pathway enrichment analyses for targets of EMO against depression

**Figure 6:**
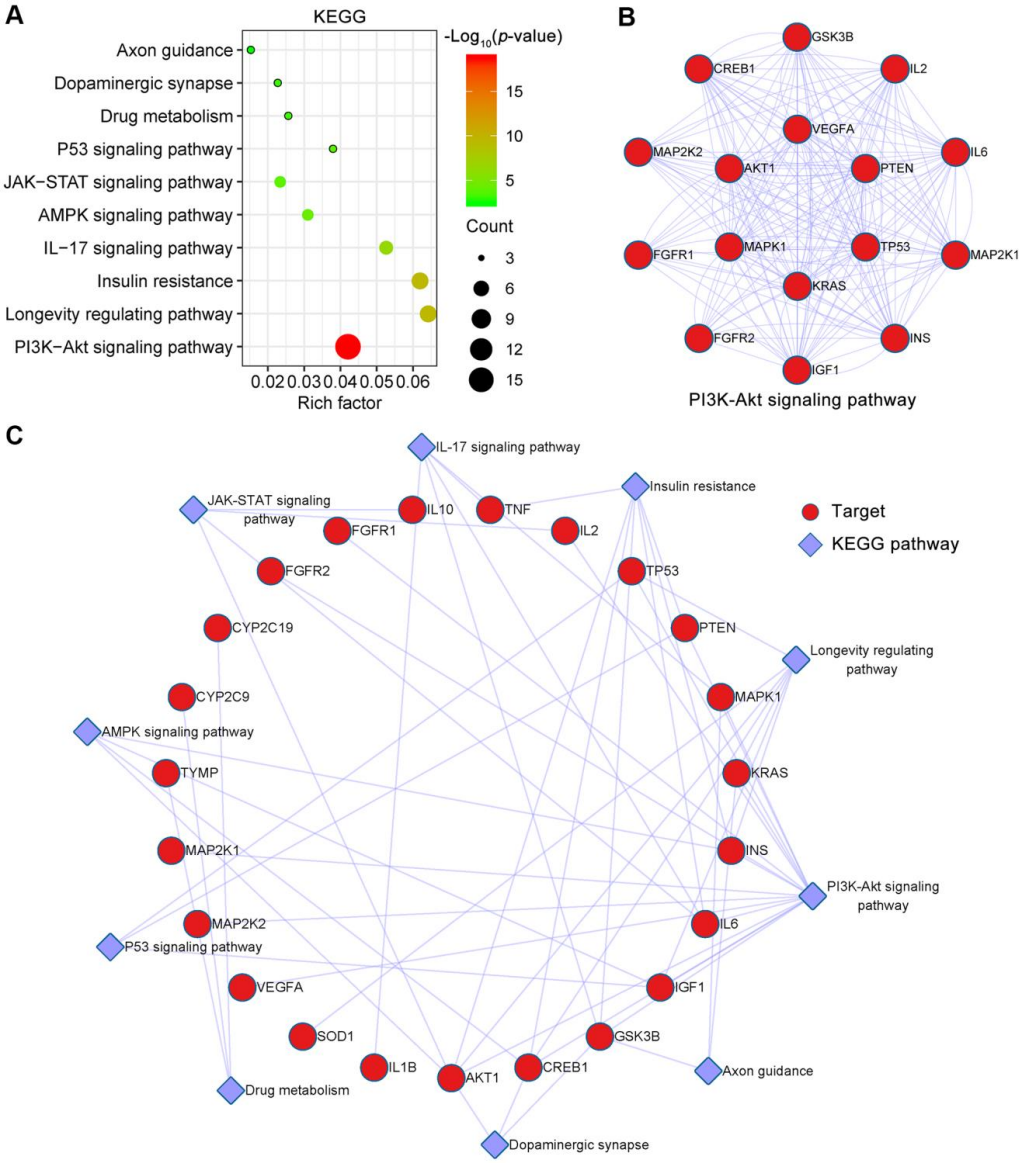
**KEGG pathway enrichment analysis of EMO against depression.** (**A**) The top 10 KEGG pathways are presented in the bubble chart. The X-axis represents the rich factor, bubble size represents the count of targets enriched in terms and the color represents the *p* value. (**B**) The PPI network of targets involved in the PI3K-Akt signaling pathway. (**C**) KEGG pathway-target network diagram of EMO treatment of depression. Red nodes represent target proteins and blue diamond nodes represent enriched KEGG pathways.

To identify signaling pathways associated with targets of EMO against depression, Metascape was used to enrich pathways for these 43 potential targets. KEGG pathways mainly involved PI3K-Akt signaling pathway (hsa04151), longevity regulating pathway (hsa04211), insulin resistance (hsa04931), IL-17 signaling pathway (hsa04657), AMPK signaling pathway (hsa04152) and so on (Figure 6A). Detailed information on the KEGG pathway enrichment analysis is shown in Table 2. Sixteen potential targets involved in the PI3K-Akt signaling pathway formed a complex PPI network, which included 16 nodes and 100 edges (Figure 6B). Moreover, the KEGG pathway-target network is shown in Figure 6C.

**Table 2:** KEGG pathway enrichment analysis of EMO against depression.

**Term**	**Pathway**	**Rich factor**	***p* value**	**Count**	**Symbols**
hsa04151	PI3K-Akt signaling pathway	0.04	1.84E-19	16	AKT1,CREB1,FGFR1,FGFR2,GSK3B,IGF1,IL2,IL6,INS,KRAS,MAPK1,MAP2K1,MAP2K2,PTEN,TP53,VEGFA
hsa04211	Longevity regulating pathway	0.06	3.20E-10	7	AKT1,CREB1,IGF1,INS,KRAS,SOD1,TP53
hsa04931	Insulin resistance	0.06	4.13E-10	7	AKT1,CREB1,GSK3B,IL6,INS,PTEN,TNF
hsa04657	IL-17 signaling pathway	0.05	3.52E-07	5	GSK3B,IL1B,IL6,MAPK1,TNF
hsa04152	AMPK signaling pathway	0.03	4.62E-05	4	AKT1,CREB1,IGF1,INS
hsa04630	JAK-STAT signaling pathway	0.02	1.38E-04	4	AKT1,IL2,IL6,IL10
hsa04115	P53 signaling pathway	0.04	2.46E-04	3	IGF1,PTEN,TP53
hsanan01	Drug metabolism	0.03	7.77E-04	3	CYP2C19,CYP2C9,TYMP
hsa04728	Dopaminergic synapse	0.02	1.10E-03	3	AKT1,CREB1,GSK3B
hsa04360	Axon guidance	0.02	3.39E-03	3	GSK3B,KRAS,MAPK1

### Molecular docking simulation

Molecular docking analysis was used to validate the binding of EMO to core targets, and the lowest energy docking model was selected. Delta G is defined as the binding energy based on the ensemble free energy; the larger the absolute value of Delta G is, the more stable the binding. The molecular docking results of EMO to core targets are shown in Table 3. Among these targets, EMO showed the highest binding energy to ESR1, GSK3B, AKT1 and VEGFA, and the Delta G was -7.88, -7.50, -7.44 and -7.30 kcal/mol, respectively.

**Table 3:** Molecular docking of the core target proteins with EMO.

**Target**	**PDB**	**deltaG (kcal/mol)**	**deltaGvdw**	**FullFitness (kcal/mol)**	**Energy (kcal/mol)**
ESR1	2QE4	-7.88	-42.93	-2044.10	-0.18
GSK3B	2O5K	-7.50	-46.88	-1970.56	5.53
AKT1	3O96	-7.44	-42.56	-2371.46	5.79
VEGFA	6D3O	-7.30	-42.31	-1318.42	4.85
PTEN	1D5R	-7.28	-49.21	-2243.15	13.89
INS	2QIU	-7.17	-45.86	-632.54	17.49
MAPK1	5LCK	-7.12	-43.05	-1970.85	13.09
TNF	3ALQ	-6.81	-38.30	-3618.49	6.94
IL6	1IL6	-6.73	-34.96	-1453.89	13.41
TP53	1KZY	-6.68	-34.52	-1936.80	11.21

**Figure 7:**
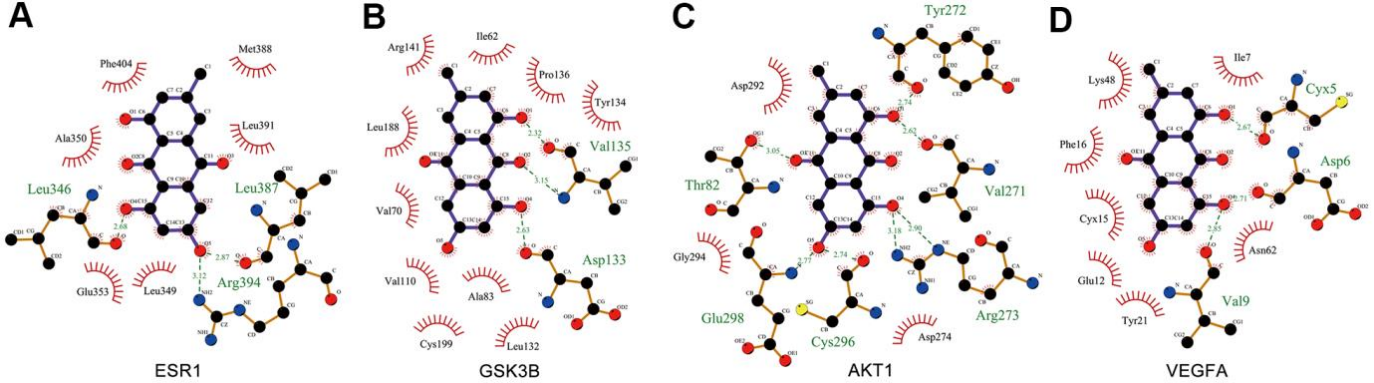
**Interaction between EMO and core targets by docking analysis.** LigPlus schematic 2D representation of EMO-core target interactions (**A**–**D**). Hydrogen bonds between EMO and the core target are represented by green dashed lines. The amino acid residues of the core target interacting with EMO are shown as brown sticks and labeled in green.

Ligand–protein interactions were calculated using LigPlot [30]. Figure 7 demonstrates that EMO binds tightly in the ESR1, GSK3B, AKT1 and VEGFA binding pockets and is stabilized by hydrogen bond interactions. Specifically, EMO formed potential interactions with residues Arg394, Leu346 and Leu387 of ESR1 through hydrogen bonds (Figure 7A). The distances between EMO and Arg394, Leu346, Leu387 were 3.12, 2.68 and 2.87 Å, respectively. Moreover, EMO formed potential interactions with residues Asp133 and Val135 of GSK3B through hydrogen bonds (Figure 7B). AKT1 is a key mediator of the PI3K-Akt pathway. EMO bound with AKT1 by forming seven hydrogen bonds at Arg273, Cys296, Glu298, Thr82, Tyr272 and Val271 residues (Figure 7C). In addition, EMO also formed potential interactions with residues Asp6, Cyx5 and Val9 of VEGFA through hydrogen bonds (Figure 7D). These findings suggested that EMO has significant binding to core targets.

### EMO reduced CUMS induced microglial activation and neuroinflammation

**Figure 8:**
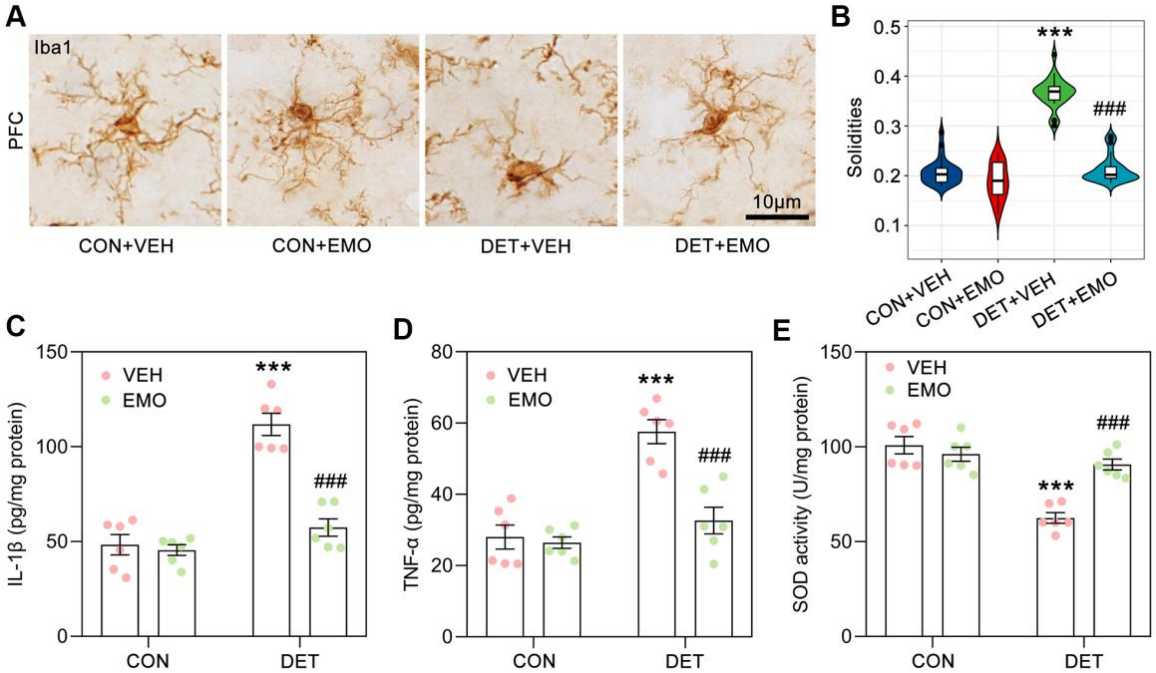
**EMO treatment significantly reduced CUMS induced microglial activation and neuroinflammation.** (**A**) Microglia in the PFC were identified by Iba1 immunohistochemical staining (bar = 10 μm). (**B**) The solidities of microglia were calculated to evaluate the activation of microglia (n = 3, 8-9 slices/group). Levels of IL-1β (**C**) and TNF-α (**D**) in the prefrontal cortex (PFC) were detected by ELISA (pg/mg protein, n = 6). The level of SOD activity (**E**) in the PFC was measured by ELISA (U/mg protein, n = 6). Data were expressed as the means ± SEM. *** *p* < 0.001 DET+VEH vs CON+VEH. ### *p* < 0.001 DET+EMO vs DET+VEH.

Neuroinflammation is related to the pathogenesis of depression, and microglial activation is one of the characteristics of neuroinflammation [31, 32]. Microglial are significantly activated in the brains of depressive suicide victims [33]. In the present study, many anti-depressant targets of EMO against depression were associated with the inflammatory response such as IL6, TNF, IL10, CRP, IL1B, CTLA4, ALB and IL2. GO analysis also revealed significantly enriched biological processes associated with regulation of inflammatory response (GO:0050727) and response to lipopolysaccharide (GO:0032496) (Figure 5A). Here, morphological changes in microglia in the prefrontal cortex (PFC) were examined by Iba1-based immunohistochemical staining (Figure 8A) and quantified by solidity (the ratio between the object area and the total area of the convex hull) [34]. The solidity of DET+VEH rats (0.37 ± 0.01) was significantly increased compared with that of CON+VEH rats (0.21 ± 0.01). However, the solidity of DET+EMO rats (0.22 ± 0.01) treated for 2 weeks with EMO significantly decreased compared with DET+VEH rats (Figure 8B).

We further detected the cytokine levels in the PFC by ELISA, the DET+VEH rats had higher levels of interleukin-1β (IL-1β, 111.8 ± 5.9 pg/mg/protein) and tumor necrosis factor α (TNF-α, 57.6 ± 3.4 pg/mg/protein) than the CON+VEH rats (IL-1β, 48.3 ± 5.3 pg/mg/protein; TNF-α, 28 ± 3.4 pg/mg/protein), while DET+EMO rats had lower levels of IL-1β (57.4 ± 4.6 pg/mg/protein) and TNF-α (32.6 ± 3.7 pg/mg/protein) (Figure 8C, 8D). The superoxide dismutase (SOD) activity of the PFC in the DET+VEH rats (62.5 ± 2.8 U/mg protein) was significantly decreased compared with that in CON+VEH rats (100.8 ± 4.6 U/mg protein), whereas treatment with EMO led to a significant increase (90.7 ± 2.9 U/mg protein) (Figure 8E). The above results suggested that CUMS induced neuroinflammation could be rescued by EMO treatment.

### Effects of EMO on PI3K-Akt signaling pathway associated proteins

**Figure 9:**
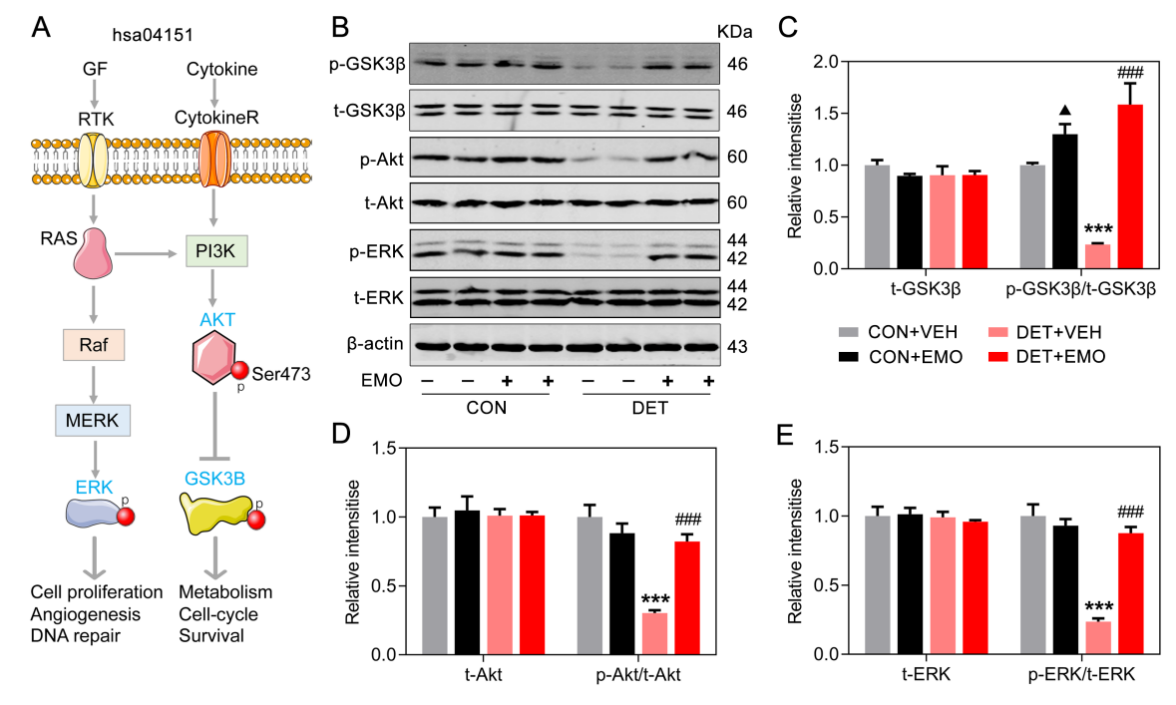
**Effects of EMO on PI3K-Akt signaling pathway associated proteins in the PFC.** (**A**) A schematic illustration of the PI3K-AKT pathway (hsa04151). (**B**–**E**) Levels of PFC total GSK3β (t-GSK3β) and phosphorylated GSK3β (p-GSK3β, Ser9), total AKT (t-AKT) and phosphorylated AKT (p-AKT, Ser473), and total ERK (t-ERK) and phosphorylated ERK (p-ERK, Thr202/Tyr204) were measured by Western blotting and quantitatively analyzed (n=4). Data were expressed as the means ± SEM. ▲ *p* < 0.05 CON+EMO vs CON+VEH. *** *p* < 0.001 DET+VEH vs CON+VEH. ### *p* < 0.001 DET+EMO vs DET+VEH.

The most significantly enriched pathway of EMO against depression was the PI3K-Akt signaling pathway (*p* = 1.84E-19), and 16 target proteins were involved (Figure 6B). More importantly, 7 out of 16 targets involved in the PI3K-Akt signaling pathway were core targets (AKT1, TP53, INS, VEGFA, IL6, MAPK1 and PTEN) (Figure 4B). As a serine/threonine protein kinase, Akt is an important kinase downstream of PI3K. There are three Akt isoforms (AKT1-3) and AKT1 is the most important subtype. GSK3β is a critical downstream target of the PI3K-Akt signaling pathway. The Ser9 position of GSK3β is phosphorylated, which causes the inactivation of GSK3β. A schematic diagram of the PI3K-Akt pathway is presented in Figure 9A. In DET+VEH rats, the levels of p-GSK3β (Ser9, 0.23 ± 0.01), p-Akt (Ser473, 0.30 ± 0.02) and p-ERK (Thr202/Tyr204, 0.23 ± 0.02) were significantly decreased (23.4%, 30.3% and 23.6% of CON+VEH rats, respectively). After EMO treatment, the levels of p-GSK3β (1.59 ± 0.21), p-Akt (0.82 ± 0.05) and p-ERK (0.88 ± 0.05) in DET+EMO rats were significantly increased. Moreover, there was no significant difference in t-GSK3β, t-Akt and t-ERK among the groups (Figure 9B–9E). The results presented above implied that EMO improves DET induced depression-related behaviors via the PI3K-Akt pathway.

### EMO treatment eliminated CUMS induced neuronal loss in the PFC

**Figure 10:**
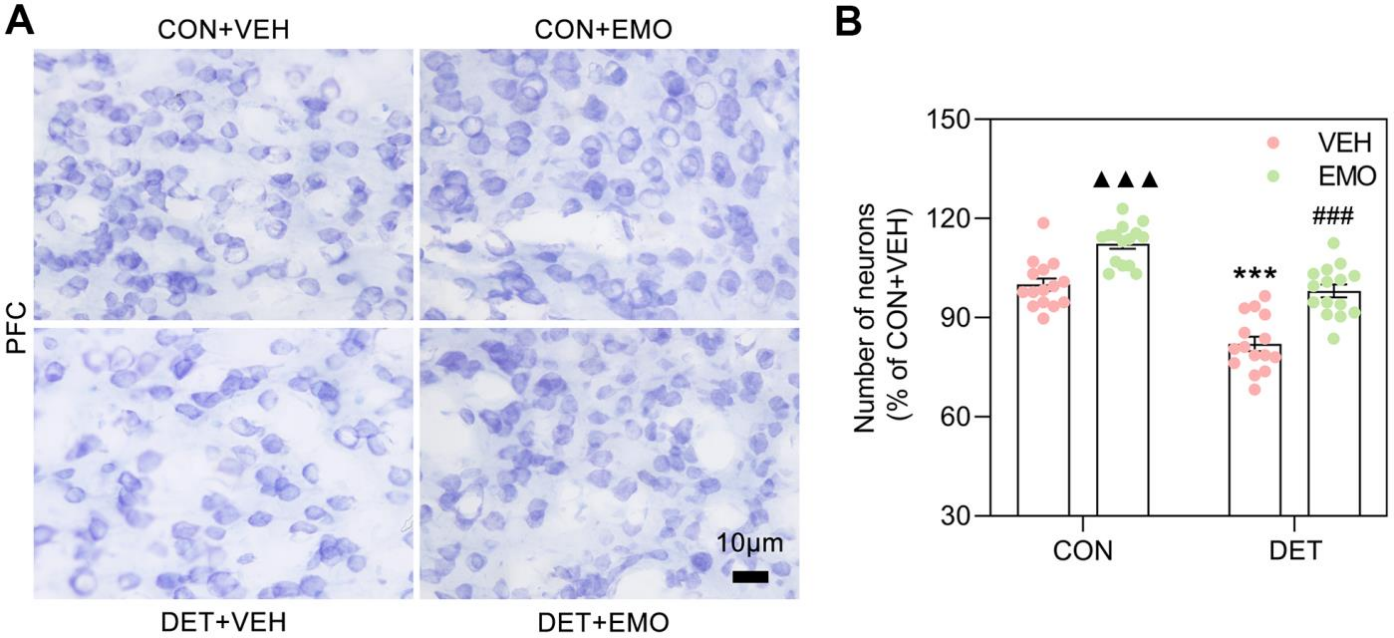
**EMO treatment reversed the loss of PFC neurons in DET+VEH rats.** (**A**, **B**) Neurons in the PFC were detected with Nissl staining (blue, bar = 10 μm) and quantified by ImageJ (n = 4, 3-4 slices/group). Data were expressed as the means ± SEM. ▲▲▲ *p* < 0.001 CON+EMO vs CON+VEH. *** *p* < 0.001 DET+VEH vs CON+VEH. ### *p* < 0.001 DET+EMO vs DET+VEH.

Nissl staining is a widely used method to identify neurons. The number of neurons in the PFC was detected with Nissl staining in this study (Figure 10A). The number of PFC neurons were decreased in DET+VEH rats (approximately 82% of the CON+VEH rats), and the neuron numbers were significantly increased after 2 weeks of EMO treatment (approximately 98.07% of the CON+VEH rats) (Figure 10B). Of note, the number of PFC neurons in CON rats was also increased substantially after EMO treatment. The above results illustrate that EMO treatment could attenuate CUMS induced neuronal loss in the PFC.

## DISCUSSION

This study demonstrated that 2 weeks of oral treatment with EMO (80 mg/kg/day) could improve the depression-like behaviors of rats exposed to CUMS, as displayed by an elevated sucrose preference percentage in the SPT, an increased zone crossing in the OFT and a reduction of immobility time in the FST. It is worth noting that not all CUMS exposed rats appeared depression, and the incidences of depression were 41.54% and 46.15% in young female and male rats, respectively [19]. Regrettably, such mistakes are not uncommon, they simply consider all CUMS-treated animals appeared depression [16, 35]. In the present study, 5 weeks of CUMS exposure was performed prior to screening out depressive tendency rats (reduced the sucrose water intake by more than 20%). Depression is a complex neuropsychiatric disease with multiple targets and signaling pathways are involved. A total of 340 depression-related targets were obtained from public databases. Depression related KEGG pathways were mainly involved neuroactive ligand-receptor interaction, cAMP signaling pathway, serotonergic synapse, dopaminergic synapse, glutamatergic synapse, PI3K-Akt signaling pathway, MAPK signaling pathway, long-term potentiation, calcium signaling pathway, Alzheimer disease and soon. To elucidate the anti-depressant effect of EMO, the underlying mechanisms were comprehensively investigated using systems pharmacology.

The PFC is a key brain region involved in the pathogenesis of depressive symptoms [36]. Depression patients showed a reduced volume of the PFC, which negatively correlated with the severity of depression, length of illness, and the duration of treatment. In addition, decreased body size of neurons, the atrophy of neuronal processes, and a decreased number of synapses in the PFC were found in postmortem studies [37]. In the PFC and hippocampus of chronic stress exposed animals, the neuronal atrophy and loss were also found, which related to the reduction in the volume of these brain regions [38]. In this study, the regulation of neuron death (GO:1901214) was identified by using the MCODE algorithm, and a series of core targets were involved, such as AKT1, IL6, TNF and GSK3B. Obvious neuronal loss was also observed in the PFC of rats with CUMS induced depression, whereas in contrast, the number of neurons in the PFC were significantly increased after EMO treatment. GSK3β, an apoptosis mediator, has been revealed to play a role in many processes of cell proliferation and apoptosis [39].

The overexpression of GSK3β in neurons significantly promoted neuronal apoptosis [40]. Regulation of PFC neuronal death may be one of the mechanisms of anti-depressant effect of EMO.

Neuroinflammation is strongly associated with the pathogenesis of depression, and anti-inflammatory agents may be a useful anti-depressant therapy or an adjuvant to the currently available conventional therapies [41]. The levels of pro-inflammatory cytokines (IL-1β and IL-6) are increased in some brain regions of patients with depression [7, 42]. A previous study also revealed that elevated levels of IL-1β, IL-6 and TNF-α were related to resistance and severity of depressive symptoms [43]. Neuroinflammation-associated microglial activation is well studied in many neuropsychiatric disorders, especially in depression [32]. Activated microglia can release pro-inflammatory cytokines (IL-1β, IL-6 and TNF-α) and injure neurons. Higher levels of pro-inflammatory cytokines in depressed animals could inhibit neurotransmission and plasticity, and suppress neurogenesis [44, 45]. The knock down of IL-1β in the hippocampus alleviates lipopolysaccharide (LPS)-induced depression-like behaviors in mice [46]. Moreover, mice exposed to CUMS significantly enhanced the production of TNF-α and IL-6 in the hippocampus, and these changes could be reversed by treatment with anti-depressant therapy [47]. This suggests that anti-inflammation plays an important role in anti-depressants. A previous report showed the anti-neuroinflammatory effect of EMO in LPS-stimulated microglia through AMPK/Nrf2 signaling [48]. In this study, 43 potential targets were shared between EMO-related and depression-related targets, implicating the possible anti-depressant effect of EMO. From the PPI network, 10 core targets (AKT1, TP53, ALB, INS, VEGFA, IL6, ESR1, MAPK1, PTEN and TNF) with the most important interactions were screened out. They were significantly enriched in several pathways such as cellular response to oxidative stress (GO:0034599), regulation of cellular response to stress (GO:0080135), regulation of inflammatory response (GO:0050727) and glial cell differentiation (GO:0010001). The results of our present study also demonstrated that CUMS treatment significantly enhanced the levels of IL-1β, TNF-α, SOD activity and microglial activation in the PFC of rats, whereas oral treatment with EMO could restore these changes. Thus, the anti-depressant effect of EMO might be involved in its anti-inflammatory activity.

The PI3K-Akt signaling pathway has been implicated in regulating the anti-depressant effects and plays an essential role in glutamate uptake, glutamate receptor trafficking, and synaptic neurotransmission [49–51]. Thus, the regulation of the PI3K-Akt signaling pathway plays a critical role in depression treatment. PI3K-Akt signaling has been implicated in the etiology of mood disorders and depression [52]. The regulation of the PI3K-Akt pathway may constitute an important signaling center in the subcellular integration of synaptic neurotransmission [53]. Animal study has also reported that the PI3K-Akt pathway was related to depressive symptom [54]. In this study, the PI3K-Akt signaling pathway was the most important pathway for the effect of EMO on depression by KEGG pathway enrichment analysis, and 16 targets were involved. This pathway modulates several functions, such as regulating cell growth, inflammation, metabolism, and cell survival. AKT1 polymorphisms are associated with depression severity, anxiety symptoms and suicide attempts in patients with depressive disorder [55]. In the present study, EMO treatment augmented the ratios of p-GSK3β (Ser9)/t-GSK3β, p-Akt (Ser473)/t-Akt and p-ERK (Thr202/Tyr204)/t-ERK in the PFC of DET+EMO rats. The molecular docking results also revealed a significant binding between EMO and GSK3β or AKT1.

An important kinase downstream of PI3K is GSK3, which consists of the highly homologous GSK3α and GSK3β [56]. The expression levels of GSK3α and GSK3β were similar in the mouse brain [57], while GSK3β was dominant in the human brain [58]. The PI3K-Akt signaling pathway plays a significant role in GSK3β activity regulation, in which Akt promotes the phosphorylation of GSK3β, resulting in GSK3β inactivation [59, 60]. Plenty of evidence shows that GSK3 contributes to pathological processes in a range of psychiatric and neurological disorders [61, 62]. Compared with non-depressed subjects, GSK3β activity was increased in the postmortem ventral PFC from subjects with depression, and GSK3α activity did not change [63]. Increased activity of GSK3β is sufficient to impair mood regulation, whereas hyperactive GSK3α alone does not impair this process [64]. Increasing evidence suggests that GSK3β inhibitors are a potential therapeutic target for depression [65–67]. Decreased hippocampal GSK3β levels ameliorated depressive-like behaviors, such as immobility times in both the FST and tail suspension tests [68]. Conversely, GSK3β overexpression in the nucleus accumbens induced a depression-like behaviors [69]. Selective GSK3β inhibitors such as LY2090314, AR-A014418 could play an anti-depressant effect [66, 67]. Thus, GSK3β may play more important roles in depression than GSK3α.

EMO plays an anti-depressant role through a multi-target approach (including GSK3β and AKT1) compared with a single GSK3β inhibitor. Moreover, the PI3K-Akt signaling pathway also play a critical role on the neuroprotection and inhibiting apoptosis via enhancing the expression of SODs [70]. EMO treatment significantly improved the SOD activity of the PFC of CUMS exposed rats in this study. PI3K-Akt signaling is a primary upstream element of the NF-κB signaling pathway. Notably, PI3K-Akt signaling pathway plays an essential role in microglial activation by stimulating NF-κB activity [71], followed by an increase in the release of inflammatory cytokines, such as IL-6, IL-1β, IL-12 and TNF-α [72–74]. These results indicate that the anti-depressant effect of EMO against CUMS-induced behaviors is directly related to the PI3K-Akt signaling pathway and its downstream neuroinflammation.

The gut-brain axis connects emotional and cognitive brain centers with gastrointestinal function and is associated with psychiatric disorders [75]. Depression affects the stability of the microbiota. Compared with healthy controls, the levels of Bacteroidetes, Proteobacteria, and Actinobacteria were strongly elevated in patients with major depressive disorder, whereas the levels of Firmicutes was significantly reduced [76]. A recent study discovered that a change in the gut microbiota brought about by chronic stress can lead to depressive-like behaviors in CUMS mice [77]. Moreover, leaky gut can cause translocation of LPS from the gut into the circulation. In turn, LPS activates various immune cells, leading to increased secretion of pro-inflammatory cytokines and systemic low-grade inflammation [78, 79]. EMO plays an anti-depressant role through gastrointestinal digestion and absorption. Several studies have reported an important role of EMO in gut microbiota modulation [80, 81]. At present, there are few reports on the effect of EMO on the relationship between the gastrointestinal system and central nervous system. Therefore, investigating the relevance of the effect of EMO on the brain and gastrointestinal system may provide a new direction for upcoming research.

## CONCLUSIONS

In this study, EMO treatment for 2 weeks can significantly improve CUMS induced depression-related behaviors. We further employed a systems pharmacology strategy and molecular docking were used to uncover the multi-target mechanisms of EMO in depression treatment. A total of 43 targets of EMO against depression were screened out. PI3K-Akt signaling pathway and its downstream neuroinflammation and neuronal loss plays an important role in the anti-depressant effect of EMO. Although more studies are needed to confirm the current results, for the first time, the mechanisms of multi-target synergistic anti-depressant of EMO have been explored in a systemic approach. Taken together, our findings might provide a theoretical basis for the application of EMO as a therapeutic for depression.

## MATERIALS AND METHODS

### Drugs and antibodies

Emodin (purity ≥ 98%, CAS# 518-82-1) was from Shanghai Base Industry (Shanghai, China). Carboxymethylcellulose sodium (CMC-Na, Cat# 30036365) was provided by Sinopharm Chemical Reagent Co. Ltd. (Shanghai, China). EMO was dissolved in 0.5% CMC-Na before use. All sandwich enzyme-linked immunosorbent assay (ELISA) kits such as for IL-1β (Cat# E-EL-R0012c), TNF-α (Cat# E-EL-R2856c) and SOD (Cat# E-BC-K020-M) were from Elabscience Biotechnology (Wuhan, China). Anti-Iba1 (Cat# 019-19741, 1:200) antibody was purchased from Wako (Osaka, Japan). Anti-β-actin (Cat# 20536-1-AP, 1:5000) antibody was obtained from Proteintech (Chicago, USA). Total glycogen synthase kinase 3β (t-GSK3β, Cat# 5676S) and phosphorylated GSK3β (p-GSK3β, Ser9; Cat# 9322S), total Akt (t-Akt, Cat# 9272) and phosphorylated Akt (p-Akt, Ser473; Cat# 4058), and total MAPK ERK1/2 (t-ERK, Cat# 4695) and phosphorylated ERK (p-ERK, Thr202/Tyr204; Cat# 4370) antibodies were purchased from Cell Signaling (Danvers, MA, USA) and diluted 1:1000 for Western blotting. Anti-rabbit (Cat# 926-32210) or anti-mouse IgG (Cat# 926-32211) conjugated to IRDye® 800 CW used in Western blotting, was purchased from Li-Cor Bioscience (Lincoln, NE, USA).

### Collection of depression-related targets

Disease-related targets were screened by the GeneCards database (https://www.genecards.org/), TTD (http://db.idrblab.net/ttd/) [22] and the Rat Genome Database (https://rgd.mcw.edu) using “depression” as a keyword, and duplicate targets were removed using Microsoft Excel software (version 2019, Microsoft Corporation, Redmond, USA). The organism was set to *Homo sapiens* in the Rat Genome Database. Specifically, the top 300 relevance score target genes were selected for further study in the GeneCards database.

### ADME evaluation

Lipinski’s rule of five was used to assess the *in vivo* absorption abilities of the designed compounds [82, 83]. Specifically, Lipinski’s rule of five includes the following: MW < 500, Hdon ≤ 5, Hacc ≤ 10, LogP ≤ 5 and Rbon ≤ 10. The SwissADME web tool (http://www.swissadme.ch) [24, 25] was used to evaluate the ADME of the compounds. Moreover, the TPSA, LogS and Log Kp were also measured.

### Screening of potential targets of EMO against depression

Potential targets of EMO were collected from the PALM-IST (http://www.hpppi.iicb.res.in/ctm/) [28] and validated through literature scanning in the PubMed database (https://pubmed.ncbi.nlm.nih.gov/). The intersection of EMO-related targets and depression-related targets was analyzed by Venny 2.1 (https://bioinfogp.cnb.csic.es/tools/venny/index.html), and the common targets were the anti-depressant target of EMO. In addition, potential target proteins were categorized by the Panther classification system (http://pantherdb.org/) [84].

### PPI network construction and the screening of core targets

The PPI network was constructed by using the STRING database (https://string-db.org/) [29]. The organism was limited to *Homo sapiens*, and only the minimum required interaction score > 0.4 was chosen as significant. The PPI network comprises nodes, which represent a target protein, and edges, which represent protein-protein interactions. The thickness of the edges represents the combined score. Degree represents the number of other nodes directly connected to a node. The higher the value of degree, the more important the node becomes. The core targets were identified through network analysis using Cytoscape software (v.3.7.1) [85] and its plugin (Network Analysis). In this study, the top 10 target proteins were selected and identified as core targets ranked by degree.

### GO and the KEGG pathway enrichment analysis

GO biological process and KEGG pathway enrichment analyses were performed using Metascape (https://metascape.org/gp) [23]. The threshold value was set as *p* < 0.01, minimum count 3, and an enrichment factor > 1.5. Moreover, the MCODE algorithm has been applied to identify highly interconnected clusters and the criteria were set as follows: degree cutoff = 2, node score cutoff = 0.2, k-core = 2, and max depth = 100. The top 20 enriched terms were visualized using an online tool (http://www.bioinformatics.com.cn). Based on the results of KEGG pathway enrichment analysis, a KEGG pathway-target network diagram of EMO treatment of depression was created by Cytoscape software.

### Molecular docking of core targets

To validate the binding of EMO to predicted core targets, the 3D molecular structure of EMO was downloaded from the PubChem database (https://pubchem.ncbi.nlm.nih.gov/). Structure files of target proteins were obtained from the RCSB Protein Data Bank (PDB database, http://www.rcsb.org/) [86]. The SwissDock (http://www.swissdock.ch/docking) [87] was used for molecular docking calculations. The interaction of residues between EMO and core target was analyzed by LigPlot (https://www.ebi.ac.uk/thornton-srv/software/LIGPLOT/) [30].

### Animals and treatments

Male Sprague-Dawley rats (8-week-old, n = 88) were from the Experimental Animal Central of Tongji Medical College, Huazhong University of Science and Technology (License No. SCXK-E 2016-0009). Rats were maintained in 12-hour-light/-dark cycle. The animal house was kept at a constant relative humidity (55 ± 15%) and room temperature (22 ± 2° C). Ethics approval was received from the Animal Care and Use Committee of Huazhong University of Science and Technology (Ethics approval number: 2019-s1845).

The CUMS procedure was carried out as previously reported [19–21]. Eighty-eight rats were randomized by Microsoft Excel software table = Rand () function. Control rats (CON rats, n = 24) were group housed 4-5 per cage, while the CUMS-treated rats (CUMS rats, n = 64) were housed individually. All animals underwent the SPT after 5 weeks of CUMS. Depressive tendency rats (DET, n = 30) were defined as a more than 20% decrease in sucrose water intake compared with before the experiment, and next divided randomly into two groups. Fifteen depressive tendency rats intragastric administrations of EMO at a dose of 80 mg/kg/day (DET+EMO rats), and the remaining animals were administered with an equal volume of vehicle (0.5% CMC-Na) (DET+VEH rats, n = 15). Meanwhile, CON rats were also given vehicle (CON+VEH rats, n = 12) and EMO (CON+EMO, n = 12). The dosage of EMO was referenced from previous studies [13, 16]. EMO treatment at (80 mg/kg/day) for 6 weeks did not cause hepatotoxicity in rats [88]. CUMS exposure was performed simultaneously with EMO treatment, and all rats completed the SPT, OFT and FST after two weeks (Figure 1A). CUMS exposure caused a decrease in body weight. After behavioral tests, the body weight of the animals was recorded.

### Behavior tests

### SPT, OFT and FST

The SPT is a classic method for detecting the loss of pleasure due to depression [89]. The SPT OFT and FST were carried out as previously described in this study [13, 19, 20]. Sucrose preference was calculated as follows: sucrose preference (%) = sucrose consumption/(water consumption + sucrose consumption) × 100%.

The zone crossing times in OFT and the total duration of immobility in FST were recorded.

### Western blotting and ELISA

Rats were sacrificed under isoflurane (RWD Life Science; Cat# R510-22) anesthesia. The brain tissues of PFC were rapidly harvested and homogenized on ice. Western blotting was performed as previously described [13, 19, 20]. The quantification of the Western blot was conducted using ImageJ (NIH, Bethesda, MD, USA).

The levels of IL-1β, TNF-α and SOD in the PFC were detected using ELISA kits, and the procedures were conducted strictly according to the instructions. The optical densities were measured at 450 nm using a BioTek Synergy 2 microplate reader (Winooski, VT).

### Immunohistochemical staining and Nissl staining

Animals were sacrificed through an over-dose of isoflurane. Immunohistochemical staining and Nissl staining were performed according to our previous studies [13, 20]. All images were captured by a microscope (NIKON 90i, Japan). The solidities [34] of microglia and the number of PFC neurons were calculated by ImageJ.

### Statistical analysis

Data were expressed as means ± SEM. Graphic plots were presented by GraphPad Prism (GraphPad Software, Inc., La Jolla, CA). SPSS 19.0 statistical software (SPSS, Chicago, IL, USA) was used for statistical analysis. One-way ANOVA procedure followed by Tukey’s multiple comparisons test was used for multiple comparisons. Statistical significance level was set at *p* < 0.05.
